# Commentary on: Abundance and distribution of microplastics within surface sediments of a key shellfish growing region of Canada

**DOI:** 10.1371/journal.pone.0225945

**Published:** 2019-12-11

**Authors:** Garth A. Covernton, Kieran Cox

**Affiliations:** Department of Biology, University of Victoria, Victoria, British Columbia, Canada; Centro de Investigacion Cientifica y de Educacion Superior de Ensenada Division de Fisica Aplicada, MEXICO

## Abstract

This formal comment is in response to “Abundance and distribution of microplastics within surface sediments of a key shellfish growing region of Canada” written by Kazmiruk and colleagues in 2018. This article presents microplastics concentrations in sediment, primarily microbeads, within Baynes Sound, British Columbia, which are some of the highest that have been reported anywhere in the world. The authors cite the local shellfish industry as the likely source of this high degree of contamination and present the industry as a substantial risk to the environment. However, the authors do not sufficiently justify the efficacy of their methodology, and there are several flaws which call into question the legitimacy of their findings. In this commentary, we address the microplastic abundances reported by the authors, and methodological concerns. Furthermore, we provide additional data to elucidate some of this study’s more contentious findings. Specifically, we seek to clarify the visual identification of microbeads and microfibres, and the microplastic concentration within shellfish populations, water, and sediment, within the Baynes Sound shellfish growing region.

## Introduction

In their paper, Kazmiruk *et al*. [1] state that up to 25,000 microplastic particles, primarily microbeads less than 0.65 μm in diameter, occur per kg of dry sediment in Baynes Sound, the premier shellfish growing region in British Columbia (BC), Canada. They claim that the area is “highly contaminated” with microplastics and that the main causes of this are the shellfish aquaculture industry and the Comox Estuary, which receives riverine input from adjacent towns. In making their case, however, the authors present several extraordinary and unsubstantiated findings and employ methodologies that call into question the validity of their microplastic concentration estimates. In this commentary we address the methodological errors, and the estimation of microplastic abundance. We believe that critically evaluating this work offers an opportunity to reflect on widely utilized practices within the field of microplastic research.

## Methodological errors

**Fig 1:**
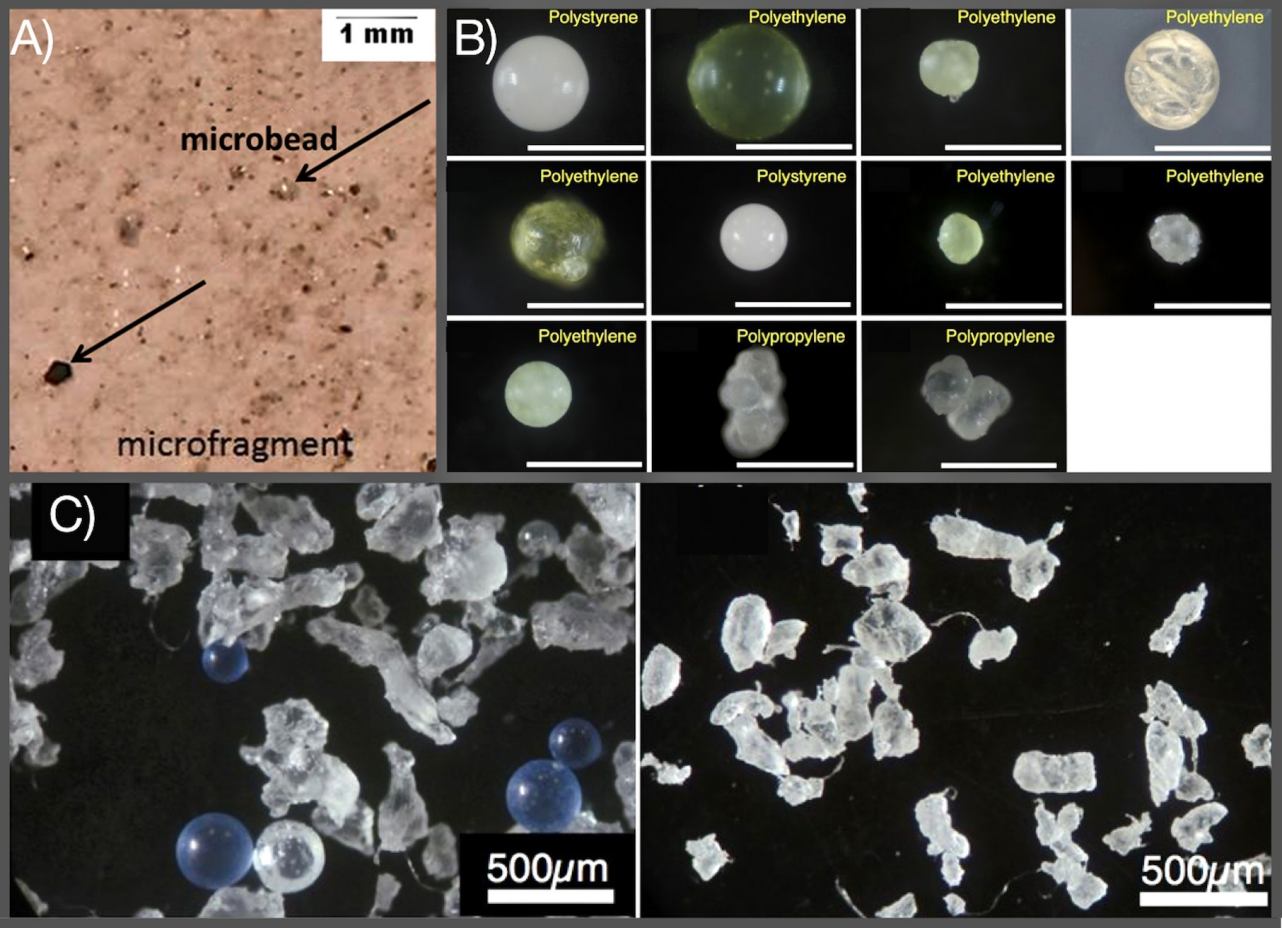
Comparison of microbeads identified by Kazmiruk *et al*. [1] and other studies. A) Figure adapted from Kazmiruk *et al*. [1] with examples of the types of microplastics recovered from sediments during their study. B) Figure adapted from Tanaka and Takada [19] indicating microbeads ingested by Japanese anchovy; white scale bars represent 500 μm. C) Figure adapted from Tanaka and Takada [19] indicating polyethylene microbeads in four brands of facial cleansers.

**Fig 2:**
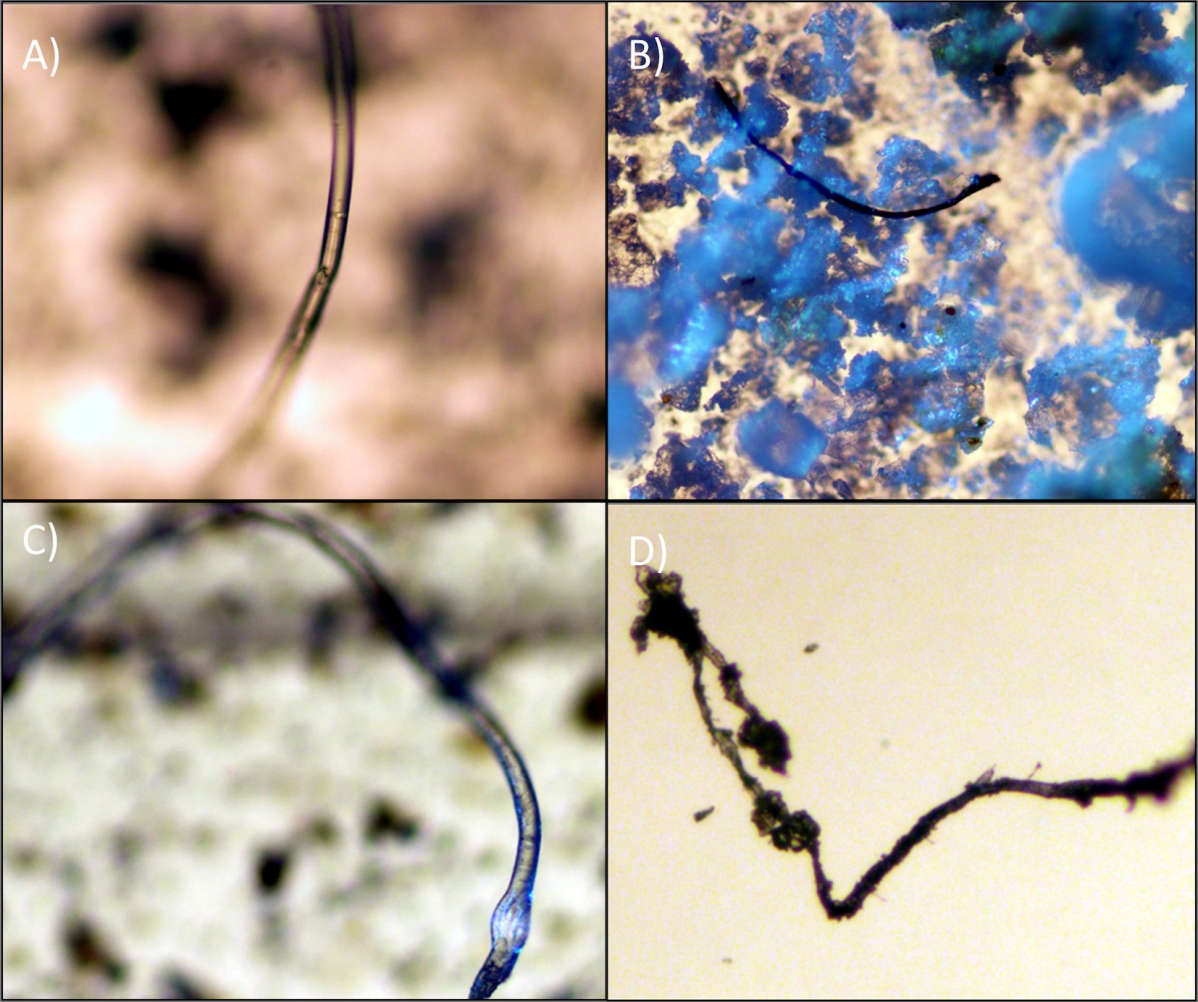
Fibres identified from southern British Columbia by Covernton *et al*. [14] and verified using FTIR spectroscopy (at 100x magnification). A) is a nylon fibre from an oyster, B) is a polyester fibre from a seawater sample, C) is a polyester fibre from a clam, and D) is a cotton fibre from a seawater sample. A) is from Baynes Sound, while the other fibres are from other regions in BC where shellfish are grown. These fibres highlight the difficulty in separating plastic from non-plastic microplastics strictly via visualization microscopy.

Visual microscopy was commonly used as the sole identification technique in the earliest studies estimating microplastic concentrations in environmental samples following the initiation of the field in 2004. More recently, the method has been questioned due to the high degree of error in identifying particles (both for false positives and false negatives), especially for those as small as the ones reported to be found by Kazmiruk *et al*. [1] (see Fig 1 for an identification of microbeads). Indeed, Dekiff *et al*. [2] isolated microplastics from beach sediment in the North Sea and concluded that the smallest size of particle that could be reliably identified by visual microscopy alone was 100 μm, and that of these only 47% could be assigned to known polymer categories, which they confirmed using thermal analysis with Pyrolysis Gas-Chromatography Mass Spectrometry. Other studies have reported that 20% of potential microplastics less than 1,000 μm in diameter in the Laurentian Great Lakes were aluminum silicate particles [3], 42% of potential non-fibrous microplastic particles from seawater were not plastic [4], and as much as 70% of visually identified microplastics were not confirmed as plastics by spectroscopic analysis [5]. Kazmiruk *et al*. [1] also identified fibres as being microplastics without confirming that they were able to successfully differentiate between plastic and non-plastic fibres, despite findings that natural and semi-synthetic fibres can make up as much as 85% of the fibre content in seawater [6] (see Fig 2 for a comparison of fibres). Kazmiruk *et al*. [1] indicate that they used automated image analysis and 40x magnification on a dissecting scope to identify their microplastic particles and state that the majority of these were less than 0.65 μm in diameter. Considering the high degree of uncertainty associated with identifying microplastic particles less than 100 μm using visual microscopy, accurately identifying particles under 0.63 μm (two orders of magnitudes less) seems implausible. Adding the automated detection process onto the visual microscopy is unlikely to increase the accuracy, given that the resolution would likely not be high enough to detect such small particles. We, and other researchers who specialize in microplastics that we consulted with, believe that the right-hand picture in Fig 2 in Kazmiruk *et al*. [1] almost certainly shows a non-plastic fibre and that it would be impossible to visually identify the particles shown in the left-hand picture as they are indistinguishable from sand, shell, or other natural particulates at the given resolution. Without chemical confirmation (*i*.*e*. spectroscopic or thermal analysis) it is impossible to confirm that all of the particles identified as microplastics in the study were indeed composed of synthetic polymers. The authors state that they used the hot hot-needle test to confirm plastic composition of particles, but from our experience, and from conversations with other microplastics researchers, it is not possible to accurately do this for the size of particle most commonly found in the study, as the width of the tip of a needle is much greater than the diameter of the particles [7,8].

In addition, Kazmiruk *et al*. [1] did not implement numerous standard methodological procedures that would validate the microplastic densities observed. These procedures are especially important within the study of microplastics, given the potential for contamination via airborne microplastics. While the authors state that they took some measures to avoid contamination (*i*.*e*. cotton laboratory coats, clean and isolated work spaces), they state that as contamination is usually in the form of fibres and that they mostly found beads, that it was not necessary to be as concerned about contamination in their study. However, they ideally would have confirmed this through the use of blanks. Procedural blanks, which involve blank samples that are subjected to identical methods as the experimental samples are a simple way of verifying the degree of background contamination that is occurring. It is good practice to include the use of procedural blanks to document the extent and nature of any background contamination that does occur. Wherever possible, the use of such blanks should also be paired with filtration of all solutions used, cleaning of all equipment with filtered water, and execution of work in a space where air flow is limited or directed away from the samples [9]. Furthermore, the authors did not attempt to remove organic matter from the samples, which can impede proper detection of microplastics and can be removed using digestion procedures such as those involving hydrogen peroxide [10]. The authors of the study in question did not discuss these and other potential sources of error, which raises concerns about the findings and interpretations of their study.

## Estimation of microplastic abundances

**Fig 3:**
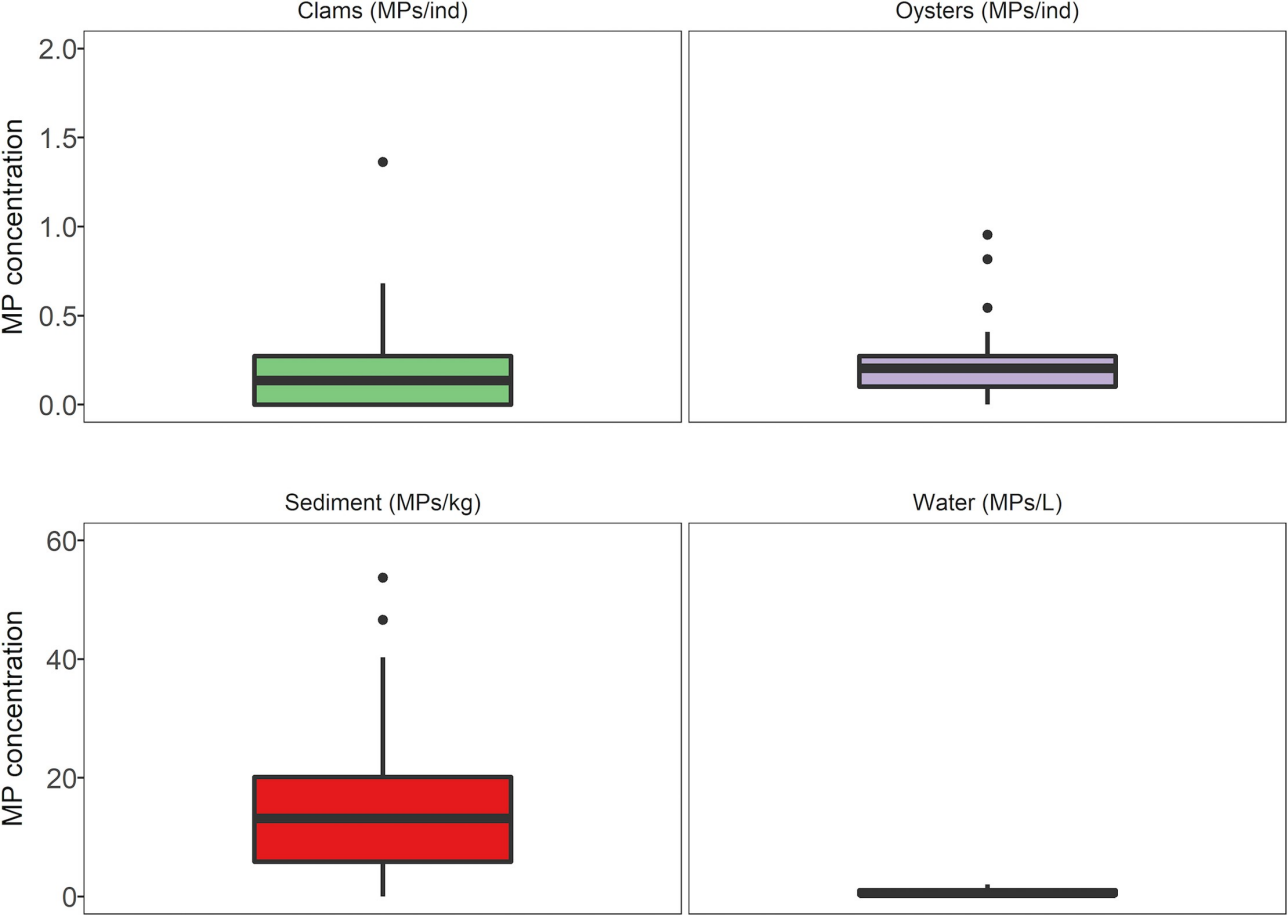
Microplastic (MP) abundance in Manila clams (*Venerupis philippinarum*), Pacific oysters (*Crassostrea gigas*), sediment, and seawater at six sites in Baynes Sound–three shellfish aquaculture sites, three non-aquaculture sites–according to unpublished work by one of the authors (Covernton *et al*. [14]). MP concentrations are expressed in terms of dry tissue weight for the bivalves, by kg dry weight for sediment, and by litre for seawater. Supporting data in S1 Dataset.

Kazmiruk *et al*. [1] state that microplastic particles, primarily microbeads, occur at a density of up to 25,000 per kg of dry sediment in Baynes Sound. If correct, this conclusion suggests that the region contains some of the highest concentrations of microplastics observed anywhere in the world. These levels seem somewhat implausible and, indeed, are not supported by the literature, including papers referenced by Kazmiruk *et al*. [1]. For example, a review by Phuong *et al*. [11] of studies relating microplastic types and concentrations in various environments reports an overall range of microplastics in sediments of 0.3–2,175 microplastic particles per kg [11–13]. Despite data limitations, it also seems that the majority of microplastics observed globally are fibres, not microbeads. In addition, Covernton *et al*. [14]found mean (± SD) of only 17.43 ± 16.13 particles kg^-1^ of dry sediment on and near shellfish farms in Baynes Sound, and 0.69 ± 0.54 particles L^-1^ in seawater, 0.17 ± 0.23 particles ind^-1^ in clams, and 0.22 ± 0.20 particles ind^-1^ in oysters, following adjustment for background contamination and an 87% visual identification error rate, which was verified by FTIR spectrometry (Fig 3). Fibres dominated all sample types. Data on microplastics in the water column in BC provided by other studies further support the hypothesis that the majority of microplastics in this area can be expected to be fibres [15,16].

Qiu et al. [17] investigated the quantity and composition of microplastics at five sediment sites in China, a country for which over 8,000,000 metric tons of plastic waste was estimated to be mismanaged in 2010, compared to Canada’s 8,000 tons [18]. Sediment microplastic concentration ranged from 5,020 to 8,720 pieces per kg of dry sediment, with FTIR spectroscopy confirming suspected plastics and identifying their composition. These values represent microplastic concentrations that are three to five-fold less than those reported by Kazmiruk *et al*. [1]. This indicates that Kazmiruk *et al*. [1] have either identified a region experiencing unparalleled contamination by microplastics in an area where plastic waste is managed comparatively well [18], or that the extremely high results are due to a misidentification error and/or a lack of chemical validation. As the study of microplastics continues to improve practices associated with the verifications of determined concentrations, it will become increasingly possible to compare microplastic densities to a global average. Reports of abundances exceeding global averages will represent areas of concern or implausible findings. Kazmiruk *et al*. [1] may be either of these cases, and without proper identification of observed plastics, it is impossible to determine.

## Supporting information

S1 Dataset (CSV)
